# Changes in global net radiative imbalance 1985–2012

**DOI:** 10.1002/2014GL060962

**Published:** 2014-08-05

**Authors:** Richard P Allan, Chunlei Liu, Norman G Loeb, Matthew D Palmer, Malcolm Roberts, Doug Smith, Pier-Luigi Vidale

**Affiliations:** 1 Department of Meteorology, University of ReadingReading, UK; 2 NASA Langley Research CenterHampton, Virginia, USA; 3 Met OfficeExeter, UK

**Keywords:** radiative flux, climate variability, satellite data, climate model, energy balance, temperature

## Abstract

Combining satellite data, atmospheric reanalyses, and climate model simulations, variability in the net downward radiative flux imbalance at the top of Earth's atmosphere (*N*) is reconstructed and linked to recent climate change. Over the 1985–1999 period mean *N* (0.34 ± 0.67 Wm^−2^) is lower than for the 2000–2012 period (0.62 ± 0.43 Wm^−2^, uncertainties at 90% confidence level) despite the slower rate of surface temperature rise since 2000. While the precise magnitude of *N* remains uncertain, the reconstruction captures interannual variability which is dominated by the eruption of Mount Pinatubo in 1991 and the El Niño Southern Oscillation. Monthly deseasonalized interannual variability in *N* generated by an ensemble of nine climate model simulations using prescribed sea surface temperature and radiative forcings and from the satellite-based reconstruction is significantly correlated (*r*∼0.6) over the 1985–2012 period.

## Key Points

- Earth's net radiative imbalance during 1985–2012 is 0.47 ±0.67 Wm^−2^
- Observed variability in Earth's radiation budget is captured by simulations
- Slower surface warming but increased radiative heating from 1985–1999 to 2000s

## 1 Introduction

The net imbalance (*N*) between absorbed shortwave radiation (ASR) and outgoing longwave radiation (OLR) at the top of Earth's atmosphere is a fundamental climate variable; it represents a nexus between changes in radiative forcings (which set the trajectory of climate change) and climate response (the magnitude of which is determined by feedbacks which may amplify or diminish climate responses) but is also influenced by unforced variability internal to the climate system [*Hansen et al.*, 2011; *Palmer et al.*, 2011; *Otto et al.*, 2013]. Its magnitude is small and difficult to measure; observationally based estimates of *N* range from around 0.5 to 1 Wm^−2^ during the 2000s with considerable interannual variability [*Hansen et al.*, 2011; *Loeb et al.*, 2012; *Trenberth et al.*, 2014]. Positive *N* indicates that energy is continuing to accumulate in the oceans, despite the apparent recent slower rates of global surface warming compared with the late twentieth century and with climate model simulations [*Fyfe et al.*, 2013a; *Watanabe et al.*, 2013].

The slower recent observed rates of global surface warming have been attributed to a combination of factors as discussed by *Trenberth and Fasullo* [2013]. These include cooling effects from natural radiative forcings [*Solomon et al.*, 2011; *Fyfe et al.*, 2013b; *Kaufmann et al.*, 2011; *Santer et al.*, 2014] and energy redistribution within the ocean due to unforced variability [*Katsman and van Oldenborgh*, 2011; *Meehl et al.*, 2013; *Kosaka and Xie*, 2013; *Watanabe et al.*, 2013; *Balmaseda et al.*, 2013; *England et al.*, 2014; *Palmer and McNeall*, 2014], although also important are sampling and measurement error of the surface temperature data [*Kennedy*, 2014; *Cowtan and Way*, 2013] and changes in stratospheric water vapor [*Solomon et al.*, 2010; *Garfinkel et al.*, 2013]; anthropogenic aerosol may also play a minor role [*Kaufmann et al.*, 2011; *Murphy*, 2013].

Quantifying, monitoring, and understanding variability in *N* is important in interpreting recent changes in global surface temperature and in constraining likely future rates of warming [e.g., *Otto et al.*, 2013]. Global coverage of ocean heat content down to 2 km depth has only recently (since around 2005) become available from a network of thousands of automated floating buoys (Argo) [e.g., *Abraham et al.*, 2013]. Combining Argo data with well-calibrated satellite data, *Loeb et al.* [2012] estimated *N* and its variability over the period 2001–2010. Aspects of this variability are questioned by *Trenberth et al.* [2014], and prior to 2000 the reliability and sampling of satellite data hamper attempts to extend this record further back in time [*Andronova et al.*, 2009; *Harries and Belotti*, 2010]. In order to overcome these inadequacies, our approach here is to exploit a combination of observations and simulations to extend the record of *N* back in time to 1985 and assess how Earth's radiative imbalance has varied during the rapid surface warming in the 1980s–1990s compared with the period since 2000.

## 2 Data Sets

**Figure 1:**
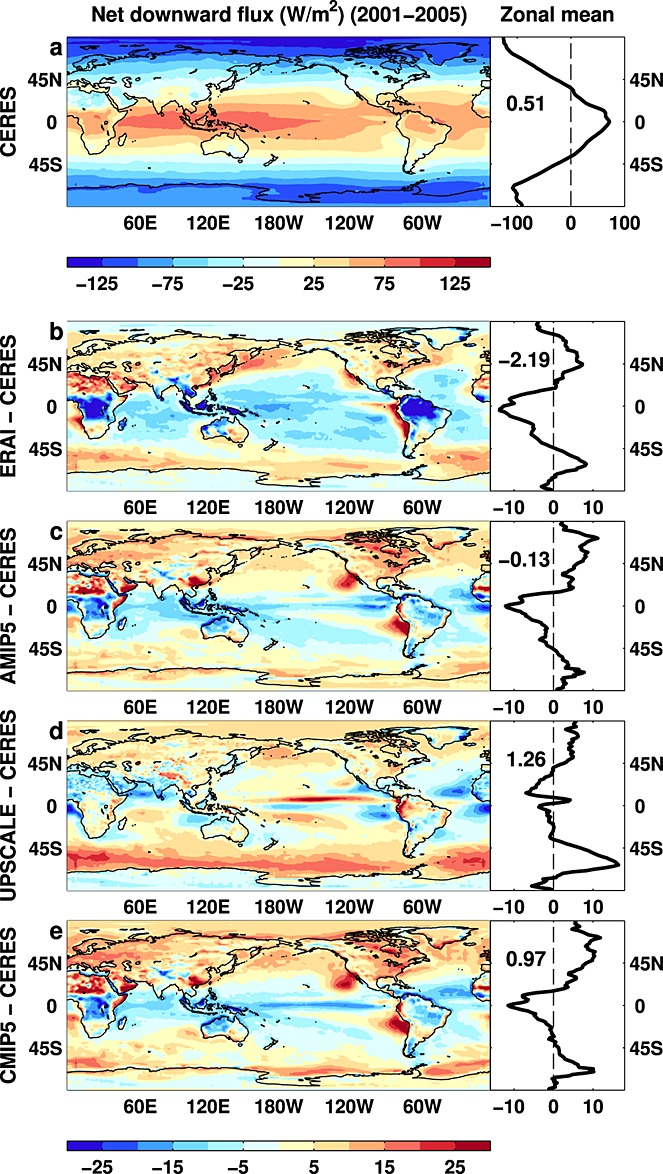
Multiannual (2001–2005) mean net downward radiative flux at the top of the atmosphere from (a) CERES observations and differences with respect to CERES for (b) ERAI, (c) AMIP5 simulations, (d) UPSCALE simulations, and (e) CMIP5 coupled simulations. Global mean values are displayed in zonal mean plots.

Central to our radiative flux reconstruction are monthly observations of top of atmosphere radiation from the Clouds and the Earth's Radiant Energy System (CERES) scanning instruments on board the Terra and Aqua satellites (Table 1). These measure outgoing total and shortwave radiances (and longwave by subtraction) which are converted, using angular dependence models, into radiative flux. ASR is calculated as the difference between incoming solar radiation from Solar Radiation and Climate Experiment and CERES outgoing shortwave radiative flux; the OLR is adjusted such that *N* is consistent with observed ocean heat uptake measured by Argo data but making major assumptions about other minor energy sinks (*N* = 0.58 ± 0.43 Wm^−2^ for July 2005 to June 2010 [*Loeb et al.*, 2012]). These Argo/CERES estimates of *N* are around 0.3 Wm^−2^ lower than those based upon multivariate ocean reanalyses which sample the entire ocean depth [*Balmaseda et al.*, 2013; *Trenberth et al.*, 2014]. Since Argo does not sample below 2000m and sampling is limited for shallow oceans, data-infilling strategies and estimates of deep ocean and non-ocean heating rates are required [*Abraham et al.*, 2013]; these uncertainties are included in the error estimates quoted above. Climatological *N*, ASR, and OLR are displayed in Figure 1a and Figures S1a and S2a in the supporting information.

**Table 1:** Observed and Simulated Data Sets

Data Set	Period	Resolution	References
CERES	2000–2012	1^∘^×1^∘^	
EBAFv2.7			*Loeb et al.* [2012]
ERBS WFOV	1985–1999	10^∘^×10^∘^	*Wielicki et al.* [2002]
Ed.3 Rev1	72days	60^∘^S–60^∘^N	*Wong et al.* [2006]
ERA Interim	1985–2012	1.5^∘^×1.5^∘^	*Dee et al.* [2011]
HadCRUT4	1985–2012	5^∘^×5^∘^	
v4.1.1.0			*Morice et al.* [2012]
CMIP5 models^a^			
CanESM2		2.77^∘^×2.81^∘^	*Arora et al.* [2011]
CNRM-CM5		1.39^∘^×1.41^∘^	*Voldoire et al.* [2012]
GISS-E2-R		2.0^∘^×2.5^∘^	*Schmidt et al.* [2014]
HadGEM2-ES		1.25^∘^×1.88^∘^	*Collins et al.* [2011]
INM-CM4		1.5^∘^×2.0^∘^	*Volodin et al.* [2010]
IPSL-CM5A-LR		1.89^∘^×3.75^∘^	*Dufresne et al.* [2013]
MIROC5		1.39^∘^×1.41^∘^	*Watanabe et al.* [2010]
MRI-CGCM3		1.11^∘^×1.13^∘^	*Yukimoto et al.* [2012]
NorESM1-M		1.89^∘^×2.5^∘^	*Zhang et al.* [2012]
UPSCALE	1985–2011	0.35^∘^×0.23^∘^	*Mizielinski et al.* [2014]

a All CMIP5 simulations include an *amip* (1985–2008) and historical/rcp45 (1985–2012) experiment (one ensemble member each). EBAFv2.7 is version 2.7 of the Energy Balance and Filled CERES product; Ed.3 Rev 1 denotes version 3 revision 1 of the ERBS WFOV dataset; HadCRUT4 is the 4th version of the Hadley Centre/Climatic Research Unit dataset, sub version 4.1.1.0; details of the CMIP5 models are available at http://cmip-pcmdi.llnl.gov/cmip5/docs/ CMIP5_modeling_groups.pdf.

**Figure 2:**
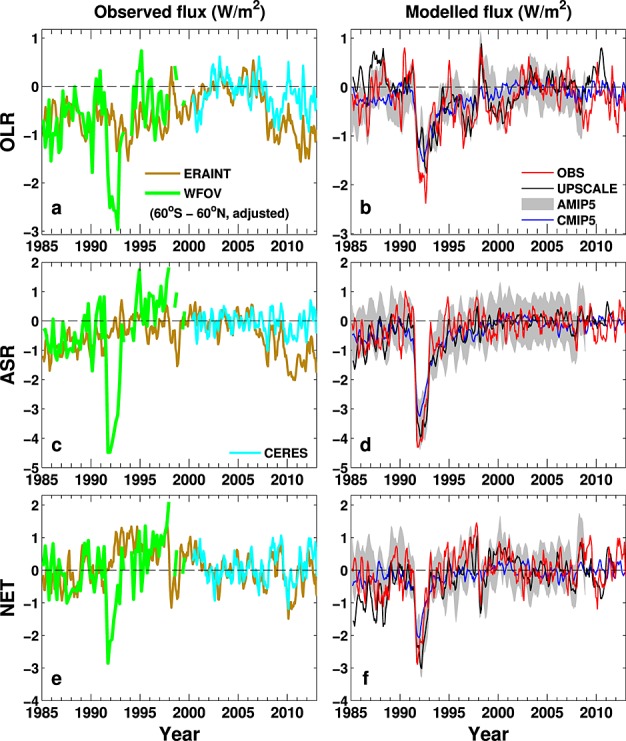
Changes in (a, c, e) observed and (b, d, f) simulated/reconstructed global mean deseasonalized anomalies (relative to the 2001–2005 period) of outgoing longwave radiation (Figures 2a and 2b), absorbed solar radiation (Figures 2c and 2d), and net downward radiation at the top of the atmosphere (Figures 2e and 2f). Three-month running means are applied. Gray shading denotes the ±1 standard deviation of the nine AMIP5 simulations. WFOV 72day mean data are deseasonalized with respect to the 1985-99 period and for clarity is adjusted so multiannual 60^∘^S–60^∘^N mean anomalies match corresponding ERAI global mean anomalies (Figures 2a, 2c, and 2e).

We also use the Earth Radiation Budget Satellite (ERBS) wide field of view (WFOV) nonscanning instrument which provides a stable, near-global record of radiative fluxes at lower spatial resolution over the period 1985–1999 (Table 1). Deseasonalized anomalies in OLR, ASR, and *N* are displayed in Figure 2.

The European Centre for Medium-range Weather Forecasts interim reanalysis (ERAI) [*Dee et al.*, 2011] applies data assimilation to a weather forecast model to provide a representation of atmospheric properties since 1979. Radiative fluxes simulated by ERAI exhibit substantial biases compared to CERES: *N* is underestimated over much of the tropics, particularly over convective regions of central Africa and Brazil (Figure 1b), due to a combination of biases in ASR (Figure S1b) and OLR (Figure S2b). However, ERAI is able to reproduce the CERES observed global monthly mean variability in *N* remarkably well, although this is not the case for OLR and ASR after 2009 (Figure 2). ERAI does not include a realistic representation of radiative forcings: for example, climatological tropospheric aerosols are prescribed and volcanic aerosols are not included, while the solar constant is unrealistically high and there is no 11year solar cycle [*Dee et al.*, 2011]. Therefore, we use ERAI data as part of the reconstruction only to provide estimates of regional changes in radiative fluxes. These are strongly constrained by the dynamical fields which are considered realistic.

We use a subset of nine climate models from the Coupled Model Intercomparison Project 5 (CMIP5) detailed in Table 1. Ensemble means are constructed from *amip* simulations (atmospheric models with prescribed observed sea surface temperature and sea ice and realistic radiative forcings, as part of the Atmospheric Modeling Intercomparison Project 5 design, AMIP5) and coupled climate model simulations which include fully circulating oceans using realistic radiative forcing up to 2005 (*historical* experiment) and projections from the *rcp*4.5 scenario after 2005 (labeled CMIP5).

A global atmospheric model (HadGEM3-A-GA3) [*Walters et al.*, 2011] in a five-member ensemble simulation at 25 km resolution [*Mizielinski et al.*, 2014] is employed to produce an extended *amip* simulation up to 2011 using the Operational Sea Surface Temperature and Sea Ice daily high-resolution Analysis (OSTIA) [*Donlon et al.*, 2012], henceforth UPSCALE. In these simulations *amip* radiative forcings were applied up to 2008 and *rcp*4.5 thereafter. The UPSCALE simulations were initialized in February 1985 with a 5year spin-up using the OSTIA forcing ending in February 1990; we do not include 1985 data in calculations to avoid any residual adjustment relating to this initialization.

The *historical* and *amip* simulations represent aerosol, although uncertainty in tropospheric aerosol radiative forcing is substantial [*Wilcox et al.*, 2013]; all models considered represent aerosol indirect effects on clouds (to varying degrees of complexity), but only the HadGEM2-ES, MIROC5, NorESM1-M, and UPSCALE simulations include the second indirect aerosol effects and the magnitude of volcanic forcing also varies between models. Additionally, increasing volcanic aerosol after 2000 is not generally accounted for [*Fyfe et al.*, 2013a; *Santer et al.*, 2014], nor is the observed negative solar radiative forcing at the end of the 2000s [*Trenberth et al.*, 2014].

The AMIP5 and CMIP5 ensemble mean simulations display similar spatial patterns of bias to each other with *N* underestimated over tropical convective regions (Figures 1c and 1e), primarily relating to ASR (Figures S2c and S2e). The UPSCALE simulation displays smaller biases in *N* (Figure 1d), although an underestimate in cloud radiative effect in the tropical west Pacific and Southern Ocean is apparent from biases in ASR and OLR (Figures S1 and S2) and are common systematic model biases [*Trenberth and Fasullo*, 2010]. The high-resolution UPSCALE simulations were not recalibrated using observations since an aim was to understand the effect of resolution upon mean climate. However, the time-varying changes exploited here are expected to be realistic. Comparison of observed and simulated variability in radiative fluxes are displayed in Figure 2and will be discussed later.

## 3 Methodology

A strategy was required to homogenize the satellite data sets. From March 2000, CERES data are used. Prior to March 2000 we reconstruct monthly mean radiative fluxes as follows (see supporting information for further details): (1) a repeating mean monthly seasonal cycle from 2001–2005 CERES data is prescribed at each grid point; (2) using ERAI data, deseasonalized radiative flux anomalies (relative to 2001–2005) are computed at each grid point, and these spatial anomalies are added to (1); and (3) a globally uniform adjustment is applied to the radiative fluxes such that 60^∘^S–60^∘^N mean deseasonalized anomalies match the WFOV time series. This approach combines the quality of the CERES data, the stability of the WFOV measurements, and the realistic circulation changes depicted by ERAI. It also reduces errors from ERAI relating to (i) spatial biases in radiative fluxes (Figure 1b), (ii) the changing observing system used in the data assimilation process [*Dee et al.*, 2011; *Allan et al.*, 2014], and (iii) unrealistic variability in radiative fluxes due to the lack of volcanic aerosol, evident in the period following the 1991 eruption of Mount Pinatubo (Figure 2). However, regional errors relating to systematic model biases and inadequate representation of tropospheric aerosols are likely to remain.

There are notable gaps in the WFOV record which may introduce unrealistic variability. First, the gap between the WFOV and CERES period (1999–2000) exhibits a systematic difference. A secondary hiatus in the WFOV record during 1993 due to a battery failure may also introduce a discontinuity in the record [*Trenberth*, 2002]. To bridge these gaps, the reconstructed fluxes prior to 2000 are adjusted such that the 2000–2001 minus 1998–1999 global mean changes agree with UPSCALE simulations; fluxes prior to 1994 are similarly adjusted based upon simulated 1994–1995 minus 1992–1993 global mean changes. The aim is to provide a plausible observation-based estimate of how radiative fluxes have changed over the period 1985–2012 (hereafter, OBS) using a combination of available satellite data and simulations.

To estimate the uncertainty in the reconstructed *N*, we combine the structural uncertainty associated with gaps in the satellite record with the CERES/Argo uncertainty estimates (±0.43 Wm^−2^ at the 90% confidence level), which includes a contribution from ocean heat content observations as well as other minor energy sinks and the CERES measurements [*Loeb et al.*, 2012]. The additional structural uncertainty is estimated from the nine-member ensemble of AMIP5 simulations and a single UPSCALE simulation, member *xgxqe*: we compute global mean changes in *N* from 1994–1995 minus 1992–1993 and 2000–2001 minus 1998–1999 and estimate the standard error (0.12 Wm^−2^ and 0.09 Wm^−2^) which was computed as the standard deviation divided by the square root of the number of degrees of freedom, which we assume is equal to the sample size (10 simulations) minus 2. Since model differences are uncorrelated between the two periods, the standard errors are combined in quadrature (0.15 Wm^−2^). This estimate of structural uncertainty includes the influence of internal variability and differences in radiative forcing. Note that all models may underestimate variability relating to volcanic radiative forcing after 2000 [*Santer et al.*, 2014], so structural uncertainty could potentially be larger. The 90% confidence range (1.64×standard error) of 0.24 Wm^−2^ was added to the CERES/Argo uncertainty to give an uncertainty range of ±0.67 Wm^−2^ (90% confidence level) for the reconstruction (applying prior to the CERES period).

*Loeb et al.* [2012] additionally estimated an annual mean 1 standard deviation uncertainty of ±0.31 Wm^−2^ for the CERES data, while the comparable uncertainty attached to the WFOV record was estimated to be 0.3–0.4 Wm^−2^ [*Wong et al.*, 2006]. Inhomogeneity in ERAI and radiative forcing inadequacies in ERAI and the UPSCALE simulations contribute further uncertainty to the regional reconstructed radiative flux variability.

## 4 Evaluating Changes in Radiative Fluxes

Variability in OLR, ASR, and *N* from the reconstruction (OBS) and simulations are displayed in Figures 2b, 2d, and 2f as deseasonalized anomalies. Substantial differences in the simulated magnitude of *N* (Table 2) are therefore removed. Anomalies are with respect to 2001–2005, explaining the differing variability to the raw WFOV data (Figures 2a, 2c, and 2e) which used a 1985–1999 baseline. The Pinatubo eruption in 1991 generates the largest perturbation to the energy balance in the years following. El Niño Southern Oscillation (ENSO) is also linked to variations in ocean heat content and in OLR and *N* [*Loeb et al.*, 2012; *Trenberth et al.*, 2014] with warmer El Niño years corresponding to higher OLR and lower *N* (e.g., 1998).

**Table 2:** Mean Net Downward Top of Atmosphere Radiative Flux, Their Standard Deviation (SD) and Ocean Heating Rates (Wm^−2^) for Different Observed and Simulated Data Sets and Time Periods

Period	OBS	ERAI	UPSCALE	AMIP5	CMIP5
1985–1989	0.23	−1.58	1.30^a^	0.71	1.53
1990–1994	0.00	−0.94	1.20	0.01	1.06
1995–1999	0.78	−1.05	2.10	0.62	1.68
2000–2004	0.63	−1.26	2.19	0.80	1.76
2005–2009	0.63	−1.45	1.98	0.85^b^	1.66
1985–2012	0.47	−1.31	1.78^a^	0.59^b^	1.57
SD	0.54	0.50	0.62^a^	0.57^b^	0.33
		ORAS4 Ocean	Ocean Observations		
Period	OBS	Reanalysis^c^	0–700m		0–1800m
1980–1989		0.43±0.11			
1990–1999	0.39±0.67	−0.18±0.09			
2000–2009	0.63±0.43	0.84±0.08			
1983–2011			0.43^d^		
1993–2008	0.65±0.67		0.49^d^; 0.39±0.09^e^		
2005–2012	0.62±0.43		0.13^d^; 0.21±0.20^e^		0.29^d^, 0.43±0.38^f^

a UPSCALE means and standard deviation for 1986–2011.

b AMIP5 means and standard deviation for 1985–2008.

c *Balmaseda et al.* [2013] full ocean depth heating rate relative to Earth's total surface area.

d *Lyman and Johnson* [2014] 2004–2011 “robust average” which applies a representative average to infill data gaps (essentially assuming that missing data share the anomaly of the surrounding data).

e *Abraham et al.* [2013] median weighted least squares fit.

f *Loeb et al.* [2012] 2005–2010.

Correlation between the observed monthly deseasonalized variability in *N* from OBS and ensemble mean simulations from AMIP5 (*r*∼0.6, 1985–2008) and UPSCALE (*r* = 0.64, 1986–2011) are significant at the 99% confidence level when applying a two-tailed test and assuming 20 degrees of freedom. Although UPSCALE simulations were used to adjust mean radiative fluxes during the 1992–1995 and 1998–2001 periods, agreement in the remaining years over the 1985–2012 period is also good.

The CMIP5 ensemble mean also captures variability associated with radiative forcings, such as the period affected by Pinatubo aerosol, but is not designed to simulate the timing of unforced variability associated with ENSO. Negative anomalies in ASR and *N* during 1985–1986 in all data sets (including CMIP5) imply smaller increases in ocean heat content which may reflect the remaining presence of volcanic aerosol from the 1982 El Chichón eruption and other smaller volcanic eruptions (Nevado del Ruiz, Augustine, and Chikurachki; see *Vernier et al.* [2011] for details) and the minimum of the solar cycle in 1985/1986.

Table 2 documents multiannual mean *N* calculated for each data set. Note that the simulations contain systematic biases in global mean net radiative imbalance but represent realistic variability as demonstrated in Figure 2. Lowest values occur in the 1991–1993 Pinatubo period, apart from ERAI which did not apply volcanic aerosol. Compared with the 2000–2009 period, reconstructed *N* is 0.15 Wm^−2^ larger in the 1995–1999 period and 0.4 Wm^−2^ lower in the early 1985-1989 period. The increases from the late 1980s to the 2000s are captured by the UPSCALE, AMIP5, and CMIP5 ensemble mean simulations, but they do not simulate a drop in *N* from the 1995–1999 to the 2000–2009 period (Table 2). Calculating *z* scores and applying a two-tail test to annual values, *N* is significantly larger in the 2000–2009 period than the 1985–1989 period at the 90% confidence level for UPSCALE (*z* = 3.1), CMIP5 (*z* = 2.4), and the OBS reconstruction (*z* = 1.8, also applicable when replacing the 2000–2009 period with 2000–2012) but not for the AMIP5 ensemble (*z* = 0.6, applying to the shorter 2000–2008 period).

**Figure 3:**
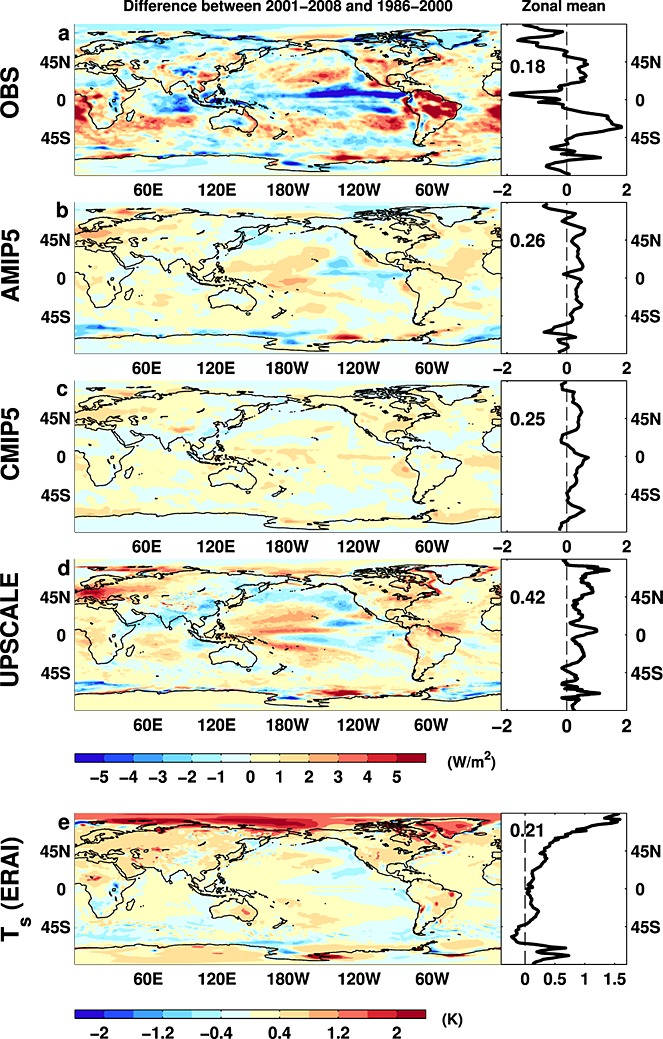
Change in net radiative flux (Wm^−2^) 2001–2008 minus 1986–2000 for (a) OBS, (b) AMIP5, (c) CMIP5, (d) UPSCALE, and (e) ERAI surface temperature changes (K). Global mean values are displayed in zonal mean plots.

To characterize the spatial signature of recent changes in Earth's radiation budget, we computed changes in *N* and surface temperature (*T*_*s*_) between the 2001–2008 and 1986–2000 periods (Figure 3; Figures S5 and S6 show OLR and ASR differences). Reconstructed regional changes in *N* are larger than simulated by the models, in part, because taking ensemble means removes some of the unforced regional circulation variability. Nevertheless, decreased *N* over the tropical east Pacific is apparent in all data sets (apart from the CMIP5 ensemble which generate their own internal ocean variability), concurrent with lower observed *T*_*s*_ (Figure 3e), and is coupled with an observed recent intensification in the Walker circulation [*L'Heureux et al.*, 2013; *Sohn et al.*, 2013] associated with changes in Pacific multidecadal variability [*Trenberth and Fasullo*, 2013] with a combination of reduced equatorial deep cloud cover and increased coverage of low-altitude cloud in the east Pacific (not shown), the details of which are model dependent (Figure S4). Conversely, generally lower *N* in the Arctic (CNRM, HadGEM2, INMCM4, MIROC5, and in particular MRI-CGCM simulations; Figure S4) are concurrent with much higher Arctic *T*_*s*_ (and higher OLR), although this is partially offset by increased ASR due to sea ice melt.

Interestingly, a majority of models (in particular, CanESM2, HadGEM2, MIROC5, NorESM1, and UPSCALE) simulate increased *N* and ASR over Europe (Figures 3b–3d and S6b–S6d), consistent with ground-based observations [*Philipona et al.*, 2009]. Since the signal is present in the CMIP5 ensemble mean, this suggests a direct influence from radiative forcings, for example, reduced anthropogenic aerosol [*Wild et al.*, 2008].

## 5 Discussion

Changes in the net downward radiative flux imbalance at the top of Earth's atmosphere (*N*) are reconstructed and analyzed over the period 1985–2012 using observations and climate model simulations. A high-resolution atmospheric model simulation is exploited to account for potential discontinuities in the satellite record in 1999/2000 and 1993. The resulting interannual monthly variability is significantly correlated with ensemble mean simulations from nine AMIP5 models (*r*∼0.6). The reconstructed net radiative imbalance over the 1985–1999 period (*N* = 0.34 ± 0.67 Wm^−2^) is lower than over the 2000–2009 period (*N* = 0.63 ± 0.43 Wm^−2^) in part due to the eruption of Mount Pinatubo in 1991, although net radiative heating in the pre-Pinatubo 1985–1989 period (*N* = 0.23 ± 0.67 Wm^−2^) is also smaller than that in the 2000s, by 0.4 Wm^−2^ (Table 2). The precise value of *N* is therefore uncertain and does not contradict previous estimates of *N* ranging from about 0.5 to 1 Wm^−2^ [*Trenberth et al.*, 2014; *Loeb et al.*, 2012; *Hansen et al.*, 2011].

The 2000–2012 mean *N* = 0.62 ± 0.43 Wm^−2^, determined by the CERES/Argo record, is lower than ocean reanalyses estimates (*N* ∼ 0.9 Wm^−2^) which sample the entire ocean depth [*Balmaseda et al.*, 2013] with an additional non-ocean heating of 0.07 Wm^−2^ estimated by *Trenberth et al.* [2014]. Curiously, the ocean heating rates during the early 2000s measured by ocean reanalyses and ocean heat content data sets reach values greater than 1 Wm^−2^ [*Trenberth et al.*, 2014], which is similar in magnitude but opposite in sign to the changes following large volcanic eruptions or large El Niño events. *Trenberth et al.* [2014] show that *N* is reduced following El Niño events, while *N* typically increases following La Niña conditions, which were not present during the 2001–2005 period (Figure S7). Such large values of *N* in the early 2000s are also not present in the CERES record [*Loeb et al.*, 2012], and this discrepancy merits further investigation.

Comparison of the WFOV and CERES satellite records with altimeter-constrained ocean heat content data indicates consistent variability in *N* [*Wong et al.*, 2006], and the reconstructed mean *N* for 1985–2012 (0.47 ± 0.67 Wm^−2^) is broadly consistent with observed heating of the upper 700m ocean of ∼0.3 Wm^−2^ [*Abraham et al.*, 2013] (1980–2012, median value of the weighted least squares linear trend in ocean heating) and 0.43 Wm^−2^ [*Lyman and Johnson*, 2014] (1983–2011, unweighted linear fits to representative mean anomalies in ocean heat content) assuming additional heating below 700m of 0.1–0.2 Wm^−2^ [*Church et al.*, 2011]. However, reconstructed *N* for the recent period (2005–2012) is strongly constrained by the 0–1800m ocean heating rate of 0.43±0.38 Wm^−2^ over the 2005–2010 period [*Loeb et al.*, 2012], which is larger than the recently updated estimate of 0–1800m ocean heating rate applying to 2004–2011 [*Lyman and Johnson*, 2014] (Table 2). These estimates are therefore sensitive to the precise set of Argo data used and the data-infilling strategies employed [*Abraham et al.*, 2013]. Additional energy sink terms, including the heating of the atmosphere, the land subsurface, and the melting and heating of ice additionally contribute [*Trenberth and Fasullo*, 2013], and simulations also indicate that substantial energy flux of ±0.1 Wm^−2^ across the 1800 m isobath are possible through internal variability alone [*Palmer and McNeall*, 2014]. Nevertheless, these sizable contributions to the uncertainty in mean *N* are not expected to degrade the realism of reconstructed variability which is informative in interpreting climate response.

An observed reduction in *N* of 0.22 Wm^−2^ between 1993–2008 and 2005–2010 based upon ocean heat content data and non-ocean heating components [*Hansen et al.*, 2011; *Abraham et al.*, 2013] is consistent with a 0.1–0.15 Wm^−2^ decrease in solar output over the 2005–2010 period [*Hansen et al.*, 2011; *Trenberth et al.*, 2014]. While reconstructed *N* decreases by 0.15 Wm^−2^ from the 1995–1999 to the 2000–2009 period, the early and late 2000s show little difference (Table 2). Mean *N* is larger in the 2000s than the late 1980s in the simulations and reconstruction despite the smaller surface warming rate in the later period, indicating an increased uptake of heat by deeper layers of the ocean [*Balmaseda et al.*, 2013]. This is also inferred by combining a simple energy balance model with reconstructions of *N* and observed surface temperature, *T*_*s*_ (see supporting information, Figures S8 and S9).

The spatial pattern of recent changes in *N* and *T*_*s*_ appears to be associated with changes in the Pacific, which is thought to be important in explaining the recent slowing in surface warming [*Kosaka and Xie*, 2013; *England et al.*, 2014]. The period since 1999 has been influenced by a number of moderate La Niña events with associated cooler east Pacific sea surface temperatures, an intensification in the Walker circulation, and reduced convection and more low cloud cover in the central and east Pacific, leading to lower *N*. This is indicative of a role for internal variability in linking radiative forcing, net radiative heating, and surface temperature changes over decadal time scales [*Katsman and van Oldenborgh*, 2011; *Meehl et al.*, 2013; *Watanabe et al.*, 2013; *Palmer and McNeall*, 2014]. Nevertheless, it is also important to note that the rate of change in *T*_*s*_ is linked to the rate of change in radiative forcings [*Hansen et al.*, 2011; *Forster et al.*, 2013]; surface cooling can occur despite positive *N* since deeper ocean heat uptake also influences the energy balance of the ocean mixed layer and therefore changes in *T*_*s*_ [*Andrews and Ringer*, 2013]. Further work is required in observing and understanding the mechanisms of ocean heat uptake and links with circulation changes [*Trenberth et al.*, 2014; *Kostov et al.*, 2014; *Mayer et al.*, 2014] before the past record of net heating, radiative forcing, and surface temperature change can be used to accurately constrain the sensitivity of climate to current changes in radiative forcing [*Otto et al.*, 2013].

## Supporting Information

Readme

## References

[b1] Abraham JP (2013). A review of global ocean temperature observations: Implications for ocean heat content estimates and climate change. Rev. Geophys.

[b2] Allan R, Liu C, Zahn M, Lavers D, Koukouvagias E, Bodas-Salcedo A (2014). Physically consistent responses of the global atmospheric hydrological cycle in models and observations. Surv. Geophys.

[b3] Andrews T, Ringer M A (2013). Cloud feedbacks, rapid adjustments and the forcing-response relationship in a transient CO_2_ reversibility scenario. J. Clim.

[b4] Andronova N, Penner J E, Wong T (2009). Observed and modeled evolution of the tropical mean radiation budget at the top of the atmosphere since 1985. J. Geophys. Res.

[b5] Arora V K, Scinocca J F, Boer G J, Christian J R, Denman K L, Flato G M, Kharin V V, Lee W G, Merryfield W J (2011). Carbon emission limits required to satisfy future representative concentration pathways of greenhouse gases. Geophys. Res. Lett.

[b6] Balmaseda M A, Trenberth K E, Källén E (2013). Distinctive climate signals in reanalysis of global ocean heat content. Geophys. Res. Lett.

[b7] Church J A, White N J, Konikow L F, Domingues C M, Cogley J G, Rignot E, Gregory J M, van den Broeke M R, Monaghan A J, Velicogna I (2011). Revisiting the Earth's sea-level and energy budgets from 1961 to 2008. Geophys. Res. Lett.

[b8] Collins WJ (2011). Development and evaluation of an Earth-system model—HadGEM2. Geosci. Model Dev. Discuss.

[b9] Cowtan K, Way R G (2013). Coverage bias in the HadCRUT4 temperature series and its impact on recent temperature trends. Q. J. R. Meteorol. Soc.

[b10] Dee DP (2011). The ERA-Interim reanalysis: Configuration and performance of the data assimilation system. Q. J. R. Meteorol. Soc.

[b11] Donlon C J, Martin M, Stark J, Roberts-Jones J, Fiedler E, Wimmer W (2012). The Operational Sea Surface Temperature and Sea Ice Analysis (OSTIA) system. Remote Sens. Environ.

[b12] Dufresne JL (2013). Climate change projections using the IPSL-CM5 Earth System Model: From CMIP3 to CMIP5. Clim. Dyn.

[b13] England M H, McGregor S, Spence P, Meehl G A, Timmermann A, Cai W, Gupta A S, McPhaden M J, Purich A, Santoso A (2014). Recent intensification of wind-driven circulation in the Pacific and the ongoing warming hiatus. Nat. Clim. Change.

[b14] Forster P M, Andrews T, Good P, Gregory J M, Jackson L S, Zelinka M (2013). Evaluating adjusted forcing and model spread for historical and future scenarios in the CMIP5 generation of climate models. J. Geophys. Res. Atmos.

[b15] Fyfe J C, Gillett N P, Zwiers F W (2013a). Overestimated global warming over the past 20 years. Nat. Clim. Change.

[b16] Fyfe J C, von Salzen K, Cole J N S, Gillett N P, Vernier J P (2013b). Surface response to stratospheric aerosol changes in a coupled atmosphere-ocean model. Geophys. Res. Lett.

[b17] Garfinkel C I, Waugh D W, Oman L D, Wang L, Hurwitz M M (2013). Temperature trends in the tropical upper troposphere and lower stratosphere: Connections with sea surface temperatures and implications for water vapor and ozone. J. Geophys. Res. Atmos.

[b18] Hansen J, Sato M, Kharecha P, von Schuc ann K (2011). Earth's energy imbalance and implications. Atmos. Chem. Phys.

[b19] Harries J E, Belotti C (2010). On the variability of the global net radiative energy balance of the nonequilibrium Earth. J. Clim.

[b20] Katsman C A, van Oldenborgh G J (2011). Tracing the upper ocean's “missing heat,”. Geophys. Res. Lett.

[b21] Kaufmann R K, Kauppi H, Mann M L, Stock J H (2011). Reconciling anthropogenic climate change with observed temperature 1998–2008. Proc. Natl. Acad. Sci. U.S.A.

[b22] Kennedy J J (2014). A review of uncertainty in in situ measurements and data sets of sea-surface temperature. Rev. Geophys.

[b23] Kosaka Y, Xie S-P (2013). Recent global-warming hiatus tied to equatorial Pacific surface cooling. Nature.

[b24] Kostov Y, Armour K C, Marshall J (2014). Impact of the Atlantic meridional overturning circulation on ocean heat storage and transient climate change. Geophys. Res. Lett.

[b25] L'Heureux M L, Lee S, Lyon B (2013). Recent multidecadal strengthening of the Walker circulation across the tropical Pacific. Nat. Clim. Change.

[b26] Loeb N G, Lyman J M, Johnson G C, Allan R P, Doelling D R, Wong T, Soden B J, Stephens G L (2012). Observed changes in top-of-the-atmosphere radiation and upper-ocean heating consistent within uncertainty. Nat. Geosci.

[b27] Lyman J M, Johnson G C (2014). Estimating global ocean heat content changes in the upper 1800 m since 1950 and the influence of climatology choice. J. Clim.

[b28] Mayer M, Haimberger L, Balmaseda M A (2014). On the energy exchange between tropical ocean basins related to ENSO. J. Clim.

[b29] Meehl G A, Hu A, Arblaster J M, Fasullo J, Trenberth K E (2013). Externally forced and internally generated decadal climate variability associated with the interdecadal Pacific oscillation. J. Clim.

[b30] Mizielinski MS (2014). High resolution global climate modelling; the upscale project, a large simulation campaign. Geosci. Model Dev. Discuss.

[b31] Morice C P, Kennedy J J, Rayner N A, Jones P D (2012). Quantifying uncertainties in global and regional temperature change using an ensemble of observational estimates: The HadCRUT4 data set. J. Geophys. Res.

[b32] Murphy D M (2013). Little net clear-sky radiative forcing from recent regional redistribution of aerosols. Nat. Geosci.

[b33] Otto A (2013). Energy budget constraints on climate response. Nat. Geosci.

[b34] Palmer M D, McNeall D J, Dunstone N J (2011). Importance of the deep ocean for estimating decadal changes in Earth's radiation balance. Geophys. Res. Lett.

[b35] Palmer M D, McNeall D J (2014). Internal variability of Earth's energy budget simulated by CMIP5 climate models. Environ. Res. Lett.

[b36] Philipona R, Behrens K, Ruckstuhl C (2009). How declining aerosols and rising greenhouse gases forced rapid warming in Europe since the 1980s. Geophys. Res. Lett.

[b37] Santer BD (2014). Volcanic contribution to decadal changes in tropospheric temperature. Nat. Geosci.

[b38] Schmidt GA (2014). Configuration and assessment of the GISS ModelE2 contributions to the CMIP5 archive. J. Adv. Model. Earth Syst.

[b39] Sohn B J, Yeh S-W, Schmetz J, Song H-J (2013). Observational evidences of Walker circulation change over the last 30 years contrasting with GCM results. Clim. Dyn.

[b40] Solomon S, Rosenlof K H, Portmann R W, Daniel J S, Davis S M, Sanford T J, Plattner G-K (2010). Contributions of stratospheric water vapor to decadal changes in the rate of global warming. Science.

[b41] Solomon S, Daniel J S, Neely R R, Vernier J P, Dutton E G, Thomason L W (2011). The persistently variable background stratospheric aerosol layer and global climate change. Science.

[b42] Trenberth K E (2002). Changes in tropical clouds and radiation. Science.

[b43] Trenberth K E, Fasullo J T (2010). Simulation of present-day and twenty-first-century energy budgets of the Southern Oceans. J. Clim.

[b44] Trenberth K E, Fasullo J T (2013). An apparent hiatus in global warming?. Earth's Future.

[b45] Trenberth K E, Fasullo J T, Balmaseda M A (2014). Earth's energy imbalance. J. Clim.

[b46] Vernier JP (2011). Major influence of tropical volcanic eruptions on the stratospheric aerosol layer during the last decade. Geophys. Res. Lett.

[b47] Voldoire A (2012). The CNRM-CM5.1 global climate model: Description and basic evaluation. Clim. Dyn.

[b48] Volodin E M, Dianskii N A, Gusev A V (2010). Simulating present-day climate with the INMCM4.0 coupled model of the atmospheric and oceanic general circulations. Izv. Atmos. Oceanic Phys.

[b49] Walters DN (2011). The Met Office Unified Model Global Atmosphere 3.0/3.1 and jules Global Land 3.0/3.1 configurations. Geosci. Model Dev. Discuss.

[b50] Watanabe M (2010). Improved climate simulation by MIROC5: Mean states, variability, and climate sensitivity. J. Clim.

[b51] Watanabe M, Kamae Y, Yoshimori M, Oka A, Sato M, Ishii M, Mochizuki T, Kimoto M (2013). Strengthening of ocean heat uptake efficiency associated with the recent climate hiatus. Geophys. Res. Lett.

[b52] Wielicki BA (2002). Evidence for large decadal variability in the tropical mean radiative energy budget. Science.

[b53] Wilcox L J, Highwood E J, Dunstone N J (2013). The influence of anthropogenic aerosol on multi-decadal variations of historical global climate. Environ. Res. Lett.

[b54] Wild M, Grieser J, Schär C (2008). Combined surface solar brightening and increasing greenhouse effect favour recent intensification of the hydrological cycle. Geophys. Res. Lett.

[b55] Wong T, Wielicki B, Lee R, Smith G, Bush K, Willis J (2006). Reexamination of the observed decadal variability of the Earth radiation budget using altitude-corrected ERBE/ERBS nonscanner WFOV data. J. Clim.

[b56] Yukimoto S (2012). A new global climate model of meteorological research institute: MRI-CGCM3—Model description and basic performance. J. Meteorol. Soc. Jpn.

[b57] Zhang Z S, Nisancioglu K, Bentsen M, Tjiputra J, Bethke I, Yan Q, Risebrobakken B, Andersson C, Jansen E (2012). Pre-industrial and mid-Pliocene simulations with NorESM-L. Geosci. Model Dev. Discuss.

