# Supplementary material for: Changes in global net radiative imbalance 1985–2012
Source: Geophys Res Lett. 2014 Aug 5;41(15):5588–97. doi: 10.1002/2014GL060962 (PMC4373161; doi:10.1002/2014GL060962)
Supplement: Supplementary file 2 — Figures S1–S9 [file grl0041-5588-sd2.pdf]

1. Supplementary information

1.1. Climatological Fluxes

Figure S1-S2 show CERES multi-annual mean absorbed solar radiation (ASR) and outgoing longwave radiation (OLR) for the 2001-2005 period. Differences in ASR and OLR, relative to CERES, are also displayed for the ERA Interim reanalysis (ERAI), AMIP5 ensemble mean, UPSCALE (HadGEM3-A-GA3) ensemble mean and CMIP5 historical experiment ensemble mean for the 2001-2005 periods (see main text for definitions of the datasets and references). A large overestimate in ASR and OLR in the west Pacific and east Indian ocean of around 20 Wm^{-2} is apparent in the UPSCALE simulations (Fig. S1d and Fig. S2d) which compensate to produce relatively small errors in N (Fig. 1d). These relate to the deficiencies in the south Asian monsoon and deep cloud in the maritime continent which are even larger in the lower resolution versions of this model. The overestimate in UPSCALE ASR in the Southern Ocean (Fig. S1d) relates to lower altitude cloud also apparent in ERAI (Fig. S1b) and is a common systematic model bias as discussed in the main text.

1.2. Reconstruction Method

Figure S3 illustrates in more detail the method used to reconstruct top of atmosphere radiative fluxes prior to the CERES record which begins in March 2000. Firstly the CERES N and OLR data are adjusted such that N=0.58 Wm^{-2} over the period July 2005-June 2010, consistent with ocean observations as detailed by *Loeb et al.* [2012]. Radiative fluxes prior to March 2000 are reconstructed as follows:

(1) 72-day 60°S-60°N mean WFOV radiative fluxes are matched in time with daily data from ERAI (Fig. S3a) and both records are deseasonalized with respect to their 1985-1999 base periods.

(2) Mean anomaly biases are computed as ERAI−WFOV; the bias is linearly interpolated in time across gaps in the WFOV record (Fig. S3b) and assumed constant after the final WFOV data point in 1999. All 72 days in each segment are assumed to have the same bias and the remaining days at the end of each year are assumed to have the same bias as the final 72-day segment of each year.

(3) The 60°S-60°N daily anomaly bias is added to the global ERAI data at each grid point over the period 1985-1999 and integrated to monthly data. Deseasonalized global monthly anomalies (relative to 2001-2005) are displayed in Fig. S3c. Since the 60°S-60°N anomalies may not be representative of global mean anomalies we examined both 60°S-60°N mean and global mean anomalies using the unadjusted ERAI dataset. Calculating the global mean anomaly as a fraction of the 60°S-60°N mean anomaly, the median scaling factor is 0.98 for OLR, 0.89 for ASR and 0.89 for N and the standard deviation of global anomalies are approximately 90% of the standard deviation of the 60°S-60°N anomalies in all cases. Therefore 60°S-60°N anomalies overestimate global mean anomalies only marginally for ASR and NET. Since we do not consider this a large effect, compared to other assumptions made, we do not scale the 60°S-60°N mean anomalies.

(4) Two-dimensional (latitude and longitude) monthly anomalies are computed for the period January 1985 to February 2000 relative to the 2001-2005 ERAI monthly climatology at each grid point. The anomalies at each grid point are added to repeating observed monthly mean seasonal

climatological fluxes, generated from CERES data over the 2001-2005 climatological period. The final global mean time series are displayed in Fig. S3d.

The culmination of these steps is ultimately to reconstruct radiative fluxes prior to the CERES record (commencing in March 2000) by applying the monthly mean climatology from the CERES dataset (2001-2005), adding the spatial deseasonalized anomalies from ERAI (relative to 2001-2005) and adjusting the global time series to match the deseasonalized (interannual) variability given by the WFOV instrument before 2000. This record is conjoined with the CERES data from March 2000 onwards.

Finally, the reconstructed data is subjected to a homogeneity adjustment as described in the main text. The reason for this is that inaccuracies may be present during the period influenced by the gap between WFOV and CERES measurements in 1999-2000 and potentially also during a gap in the WFOV record during 1993 [*Trenberth*, 2002]. Since there is no way to know the true changes during these periods, we use the following method.

We compute changes in OLR, ASR and N from the UPSCALE ensemble mean simulation over the following two periods: 1994-1995 minus 1992-1993 and 2000-2001 minus 1998-1999. The reconstructed fluxes are then adjusted prior to January 2000 and January 1994 so that changes in global mean radiative fluxes agree with UPSCALE simulations. While the UPSCALE data are climate model simulations, they use realistic radiative forcings and sea surface temperature/sea ice fields as boundary conditions and are unaffected by the changing observing systems used within the data assimilation of reanalyses such as ERAI. The UPSCALE simulations are also high spatial resolution and contain the most up-to-date parametrizations [*Walters et al.*, 2011; *Mizielinski et al.*, 2014] and so we consider that simulated flux changes are likely to be realistic

over these relatively short periods of the record although will be affected by inaccuracies in boundary conditions (sea surface temperature/sea ice and radiative forcings).

To characterize the uncertainty in N relating to internal variability of the climate system and the boundary conditions used, we also compute changes in radiative fluxes simulated by the 9 CMIP5 climate model simulations. Details of the uncertainty estimate and deseasonalized interannual anomalies of radiative flux for OLR, ASR and N are discussed in the main text and Fig. 2.

1.3. Regional changes in net flux for individual models

Figure S4 shows changes in N over the period 2001-2008 minus 1986-2000, displayed separately for the *amip* simulation from each CMIP5 climate model in Table 1. While a distinct signal of decreased N in the east Pacific and increased N in most other tropical regions is evident in Fig. 3b, the picture is more complex amongst individual models. This is partly explained by the internal atmospheric variability but also by differences in physical processes and feedbacks represented by each model simulation. In particular there are substantial differences over the eastern sub-tropical stratocumulus regions: while some models show increases in N over these regions, particularly away from coastal areas (e.g. CNRM, GISS, MIROC5) other models display decreases in N (e.g. CanESM2, HadGEM2, IPSL, MRI). It is also notable that the majority of models simulate increases in N over Europe (e.g. CanESM2, HadGEM2, IPSL, MIROC5, NorESM1) as discussed in the main text.

Changes in OLR and ASR over the period 2001-2008 minus 1986-2000 for the reconstruction and model ensembles are displayed in Figures S5-6. Increases in OLR and ASR are generally largest across northern high latitudes in all simulations and the global-average increases in OLR

and ASR are consistent across all datasets (global mean OLR increases by 0.27-0.34 Wm^{-2} and ASR increases by 0.52-0.56 Wm^{-2} for OBS, AMIP5 and CMIP5 and by 0.71 Wm^{-2} for UPSCALE). Patterns of change are consistent across the tropics for the reconstruction and *amip* experiments, reminiscent of El Niño Southern Oscillation (ENSO), with large compensation between OLR and ASR associated with spatial shifts in deep convective cloud regimes. Increases in global mean N from the 1986-2000 to the 2001-2009 period (Fig. 3, main text) are explained by increased ASR offset by smaller magnitude increases in OLR (Fig. S5-6), strongly influenced by aerosol emission and resulting cooling following the eruption of Mt. Pinatubo.

Comparison of the difference between AMIP5-CMIP5 ensemble mean radiative flux (OLR, ASR, N) helps to isolate the influence of unforced variability upon the radiative fluxes (Fig. S7). Also displayed is the Multi-variate ENSO index (MEI; *Wolter and Timlin* [1998]). The OLR is positively correlated but lagged behind MEI (El Niño precedes positive OLR anomalies) which also dominates N. The link between MEI and ASR is less clear (Fig. S7b) and there is also a residual difference between AMIP5 and CMIP5 simulations during the Pinatubo volcanic eruption which may reflect unrealistic ocean heat uptake following volcanic eruptions in the coupled simulations.

1.4. Idealized experiments with the simple energy balance climate model

To further understand the links between changes in N, T_s and ocean heating a simple global mean energy balance climate model with a two layer diffusive ocean is used [*Allan et al.*, 2014; *Watanabe et al.*, 2013]. This assumes that N is determined by changes in effective radiative forcing ΔF and a climate response ($Y\Delta T_s$) dependent upon the feedback parameter, Y ($Wm^{-2}K^{-1}$); the temperature anomaly ΔT_s in a mixed layer ocean is computed from N minus the diffused

heat flux to the deep ocean $D = k(\Delta T_s - \Delta T_D)$ where $k = 0.84\ Wm^{-2}K^{-1}$:

$$\frac{d\Delta T_s}{dt} = \frac{N - D}{C_m} = \frac{\Delta F - Y\Delta T_s - D}{C_m}; \qquad \frac{d\Delta T_D}{dt} = \frac{D}{C_D}, \tag{1}$$

with heat capacity of the mixed layer ($C_m = 4.2 \times 10^8\ JK^{-1}m^{-2}$) and deep layer ($C_D = 3.8 \times 10^9\ JK^{-1}m^{-2}$), Y varies from 1 to 2 $Wm^{-2}K^{-1}$ and ΔT_D is the deep ocean temperature anomaly. ΔF is prescribed from the IPCC Climate System Scenario Tables [*Prather et al.*, 2013] and the model is integrated from 1850. The simulation is used for illustrative purposes, to interpret changes in N and T_s using up to date estimates of ΔF, so the precise parameter values are not crucial.

Fig. S8a shows the simulated global mean ΔT_s (anomalies with respect to 1850-1870) and HadCRUT4 observations including the adjustment for missing data by *Cowtan and Way* [2013]. The simulation with less positive feedbacks ($Y = 2\ Wm^{-2}K^{-1}$) underestimates T_s during the Pinatubo period while the high climate sensitivity case ($Y = 1\ Wm^{-2}K^{-1}$) overestimates the rate of warming since 2000. Fig. S8b displays N calculated by the model and also using the same radiative forcings but prescribing ΔT_s from *Cowtan and Way* [2013] (dashed lines). Simulated N for $Y = 2\ Wm^{-2}K^{-1}$ is generally close to OBS (∼0-1 Wm^{-2}) although OBS produces a smaller drop in N following the Pinatubo eruption in 1991 and is lower than the simulations by around 1 Wm^{-2} during the 1987-88 El Niño.

We conducted simple idealized experiments with the global energy balance model described above. In particular, how can uncertainties in radiative forcing (ΔF) impact trends in surface temperature anomaly (ΔT_s) and N since 2000? To assess this we set the climate feedback parameter to a mid-range value ($Y = 1.5\ Wm^{-2}K^{-1}$) and computed changes in ΔT_s and N

using the best estimate ΔF described in the main text (black line in Fig. S8a; green line in Fig. S8b-c).

Next we generated 3 idealized ΔF scenarios from 2000:

Case 1: a continuation of the increasing trend in ΔF computed over the period 1976-1999 but avoiding the periods affected by volcanic aerosol (1982-84 and 1991-1993), signified by the thick and thin red lines in Fig. S9a.

Case 2: no change in ΔF from 2000 (cyan line in Fig. S8a)

Case 3: a decreasing trend in ΔF equal in magnitude but opposite in sign to the changes in Case 1 (blue line in Fig. S9a)

Figure S8b demonstrates that ΔT_s continues to increase in the 2000s for all cases considered apart from Case 3 where decreasing ΔF leads to relatively constant ΔT_s. Fig. S9c shows that all cases lead to a decrease in N since 2000 except Case 1 where a positive trend in ΔF leads to relatively constant N. For comparison, reconstructed N from OBS is also shown in Fig. S9c (thick black line). These idealized experiments (Fig. S9) indicate that it is difficult to reconcile the observed near-constant N and T_s during the 2000s with the radiative forcing, thereby implying a role for internal variations in the heat flux exchanged between ocean layers (D) which we now investigate.

Making the assumption that observed ΔT_s approximates the mixed layer temperature changes, we next infer changes in heat flux to the deeper layer using N from OBS ($D = N - C_m(d\Delta T_s/dt)$). The aim is not to calculate precise values but to provide an indication of the tendency in deep ocean heat flux over the period inferred from our reconstruction. Fig. S8b shows a general increase in D over the period, indicative of lower deep ocean heat flux early in the period and

greater heat flux more recently. Although this could reflect errors in the reconstruction of N used in this calculation, it is broadly consistent with conclusions based upon ocean reanalyses [*Balmaseda et al.*, 2013; *England et al.*, 2014] and highlights the potential role of ocean circulation variability in contributing to the surface warming trends on decadal time-scales. However, a more in-depth treatment, within a climate modelling framework is required to understand the roles of radiative forcing and ocean heat uptake in determining current climate variability.

In conclusion, based upon the simple energy balance model, it does not appear possible to reconcile trends in N and ΔT_s since 2000 without assuming changes in heat flux below the mixed layer that are independent of the radiative forcings for the model parameters used. Therefore the relatively stable ΔT_s since 2000 appears more likely to be explained by internal variability of the climate system than by changes in radiative forcings, although a combination of factors remains plausible. Applying experiments with atmosphere-only and fully coupled comprehensive climate model simulations is required to test this hypothesis.

References

Allan, R., C. Liu, M. Zahn, D. Lavers, E. Koukouvagias, and A. Bodas-Salcedo (2014), Physically Consistent Responses of the Global Atmospheric Hydrological Cycle in Models and Observations, *Surv. Geophys.*, *35*, doi:10.1007/s10712-012-9213-z.

Balmaseda, M. A., K. E. Trenberth, and E. Källén (2013), Distinctive climate signals in reanalysis of global ocean heat content, *Geophys. Res. Lett.*, *40*, 1754–1759, doi:10.1002/grl.50382.

Cowtan, K., and R. G. Way (2013), Coverage bias in the HadCRUT4 temperature series and its impact on recent temperature trends, *Q.J.R. Meteorol. Soc.*, doi:10.1002/qj.2297.

England, M. H., S. McGregor, P. Spence, G. A. Meehl, A. Timmermann, W. Cai, A. S. Gupta, M. J. McPhaden, A. Purich, and A. Santoso (2014), Recent intensification of wind-driven circulation in the Pacific and the ongoing warming hiatus, *Nature Clim. Change*, *4*, 222–227, doi:10.1038/nclimate2106.

Loeb, N. G., J. M. Lyman, G. C. Johnson, R. P. Allan, D. R. Doelling, T. Wong, B. J. Soden, and G. L. Stephens (2012), Observed changes in top-of-the-atmosphere radiation and upper-ocean heating consistent within uncertainty, *Nature Geosci.*, *5*, 110–113, doi:10.1038/ngeo1375.

Mizielinski, M. S., et al. (2014), High resolution global climate modelling; the upscale project, a large simulation campaign, *Geosci. Model Dev. Discuss.*, *7*, 563–591, doi:10.5194/gmdd-7-563-2014

Prather, M., et al. (2013), *Annex II: Climate System Scenario Tables*, chap. Annext II, p. 50 pp., Climate Change 2013: The Physical Science Basis. Contribution of Working Group I to the Fifth Assessment Report of the Intergovernmental Panel on Climate Change, Cambridge University Press, Cambridge, United Kingdom and New York, NY.

Trenberth, K. E. (2002), Changes in tropical clouds and radiation, *Science*, *296*, 2095a.

Walters, D. N., at al. (2011), The Met Office Unified Model Global Atmosphere 3.0/3.1 and jules Global Land 3.0/3.1 configurations, *Geosci. Model Dev. Discuss.*, *4*, 1213–1271, doi: 10.5194/gmdd-4-1213-2011.

Watanabe, M., Y. Kamae, M. Yoshimori, A. Oka, M. Sato, M. Ishii, T. Mochizuki, and M. Kimoto (2013), Strengthening of ocean heat uptake efficiency associated with the recent climate hiatus, *Geophys. Res. Lett.*, *40*, 3175–3179, doi:10.1002/grl.50541.

Wolter, K. and M. S. Timlin (1998), Measuring the strength of ENSO events: How does 1997/98 rank? *Weather*, *53*, 315–324, doi:10.1002/j.1477-8696.1998.tb06408.x.

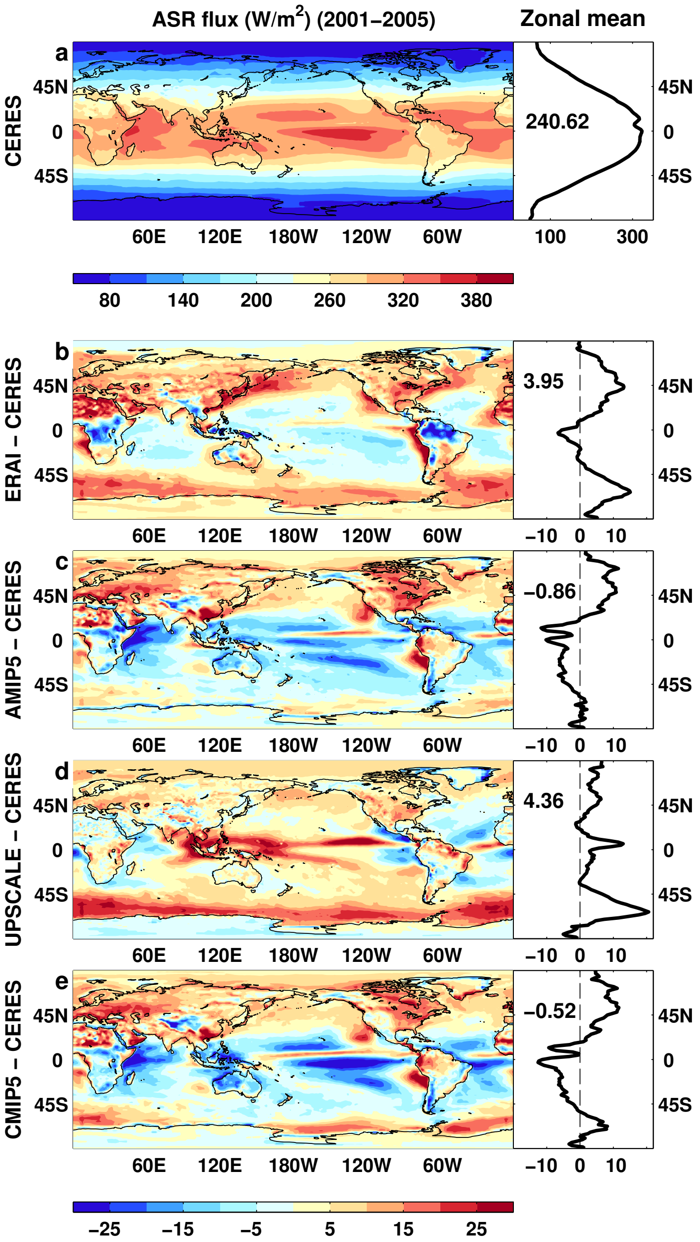

Figure S 1. Multi-annual (2001-2005) mean net absorbed solar radiation (ASR) at the top of the atmosphere from (a) CERES observations and differences with respect to CERES for (b) ERAI, (c) AMIP5 simulations, (d) UPSCALE simulations and (e) CMIP5 coupled simulations. Global mean values are displayed in zonal mean plots.

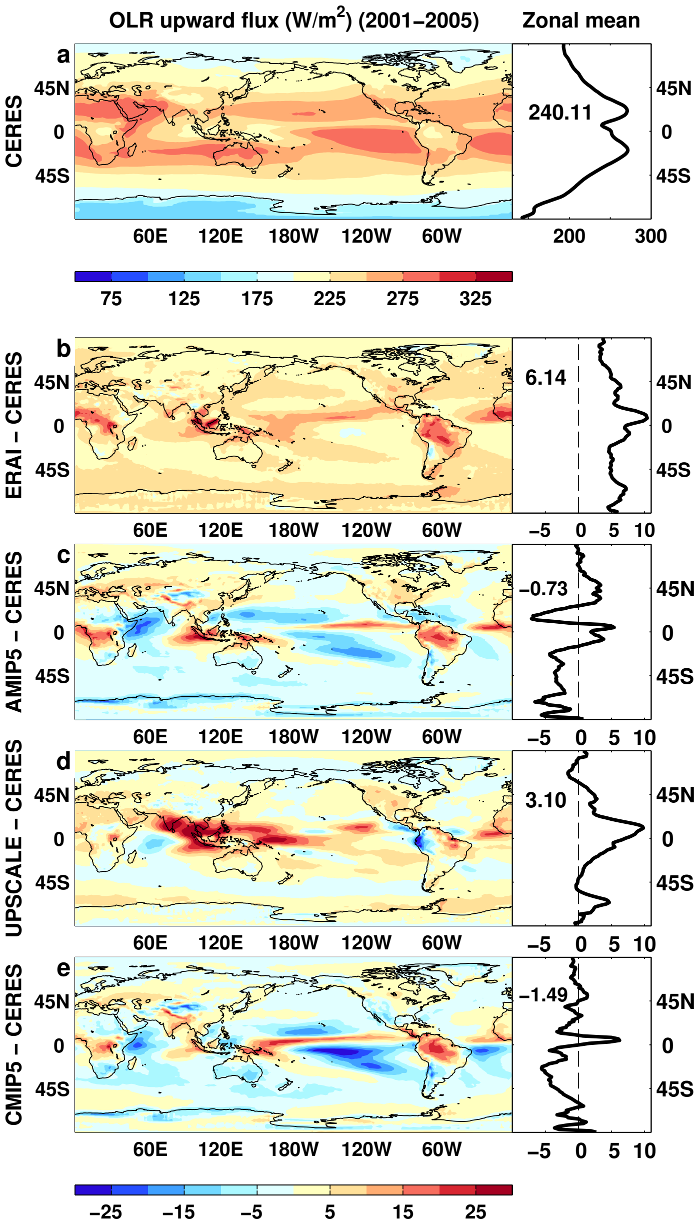

Figure S 2. Multi-annual (2001-2005) mean outgoing longwave radiation (OLR) at the top of the atmosphere from (a) CERES observations and differences with respect to CERES for (b) ERAI, (c) AMIP5 simulations, (d) UPSCALE simulations and (e) CMIP5 coupled simulations. Global mean values are displayed in zonal mean plots.

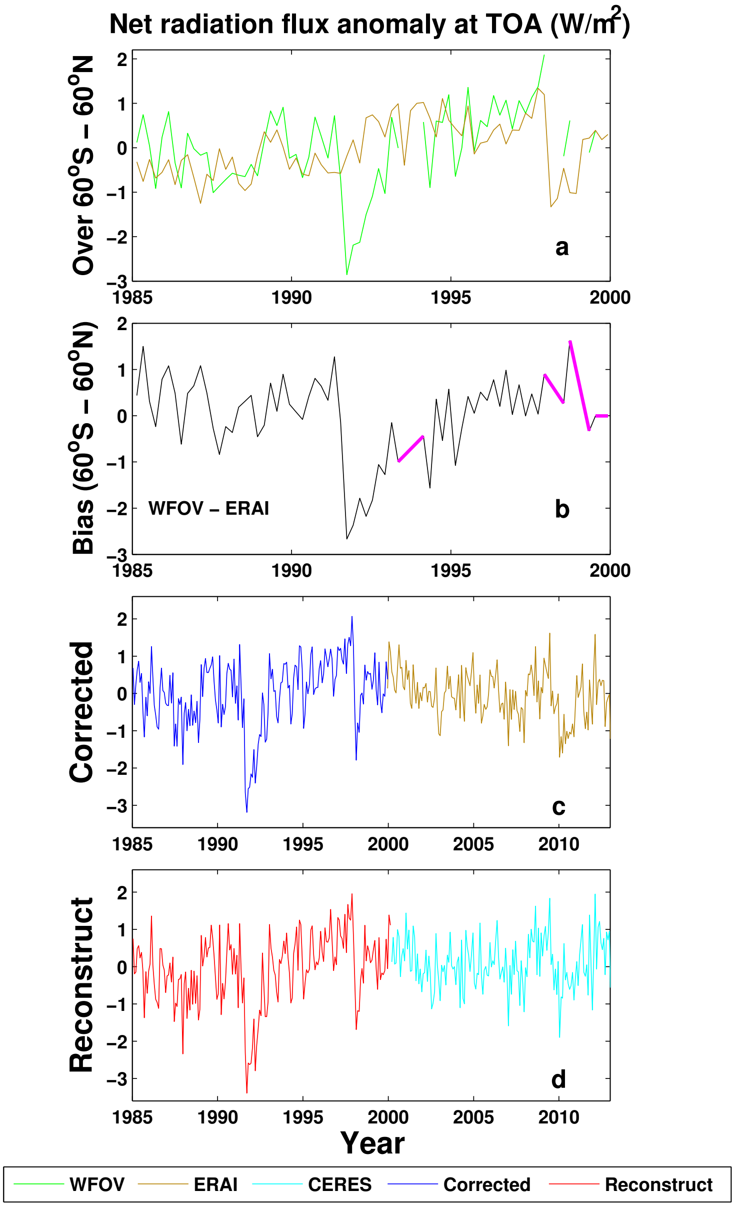

Figure S 3. Reconstruction method: (a) temporally match daily ERAI fluxes with ERBS WFOV 72-day means and deseasonalized relative to 1985-1999; (b) Compute 60°S-60°N anomaly bias and interpolate bias across gaps in record; (c) add anomalies to the daily global ERAI grid-point data and integrated to monthly (globally averaged deseasonalized anomalies shown); (d) spatial deseasonalized monthly anomalies 1985-1999 are re-calculated relative to the reference period of 2001-2005 and then added to the CERES monthly climatological means of the same reference period. Further adjustments are made for inhomogeneities in the 1993 and 1999/2000 periods as described in the main paper.

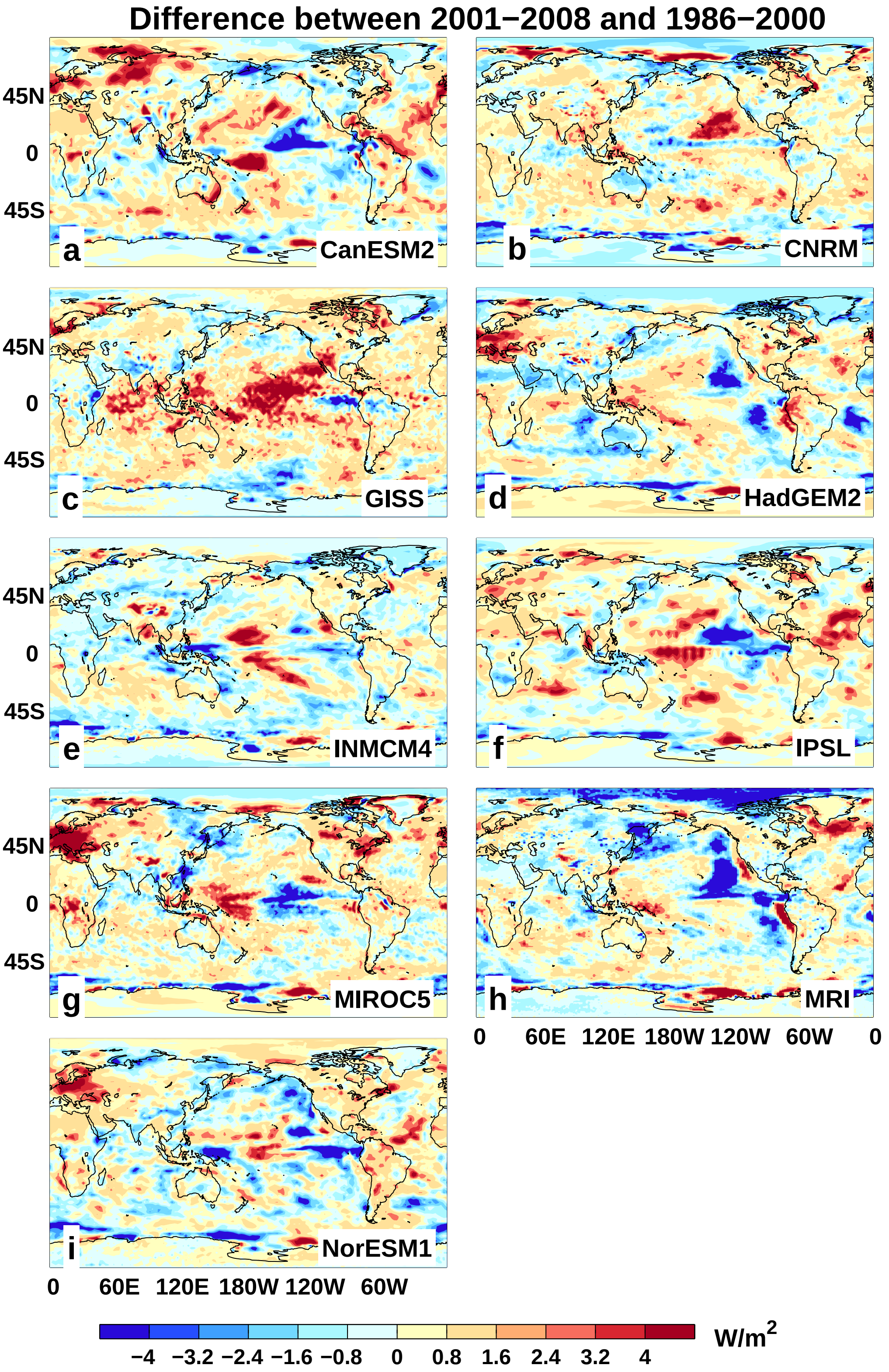

Figure S 4. Change in net radiative flux 2001−2008 minus 1986−2000 for AMIP5 models used to construct Fig. 3b.

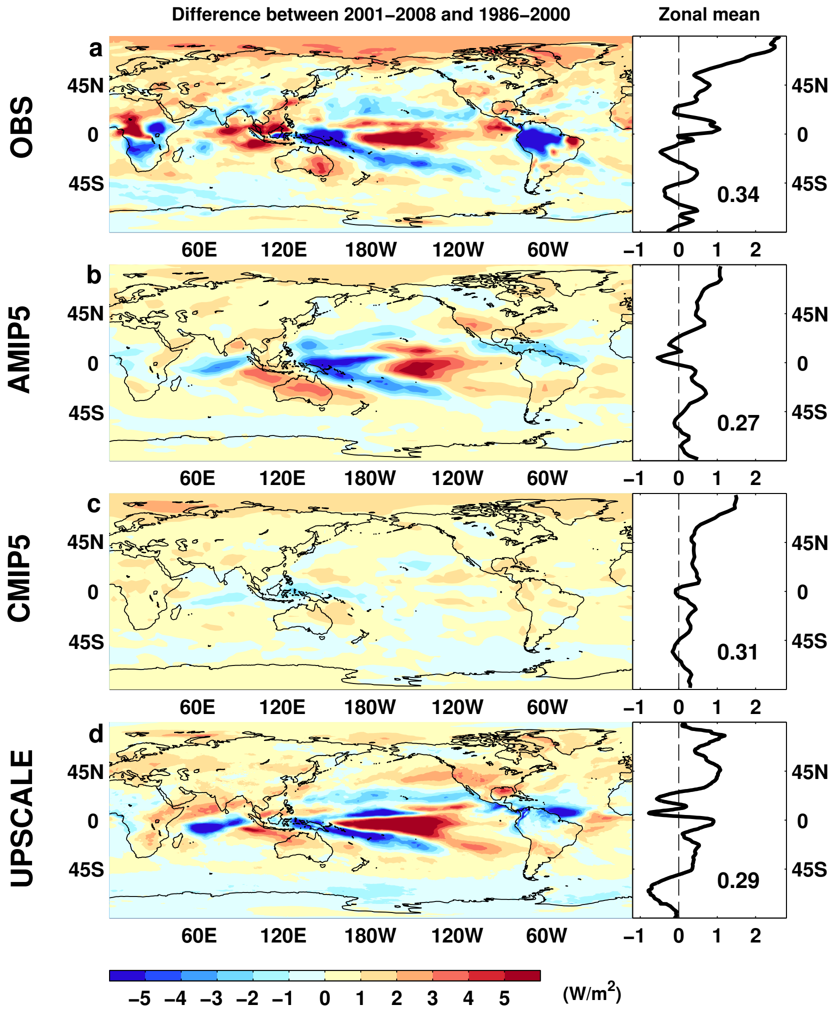

Figure S 5. Change in outgoing longwave radiation (Wm^{-2}) 2001–2008 minus 1986–2000 for (a) OBS, (b) AMIP5, (c) CMIP5 and (d) UPSCALE. Global mean values are displayed in zonal mean plots.

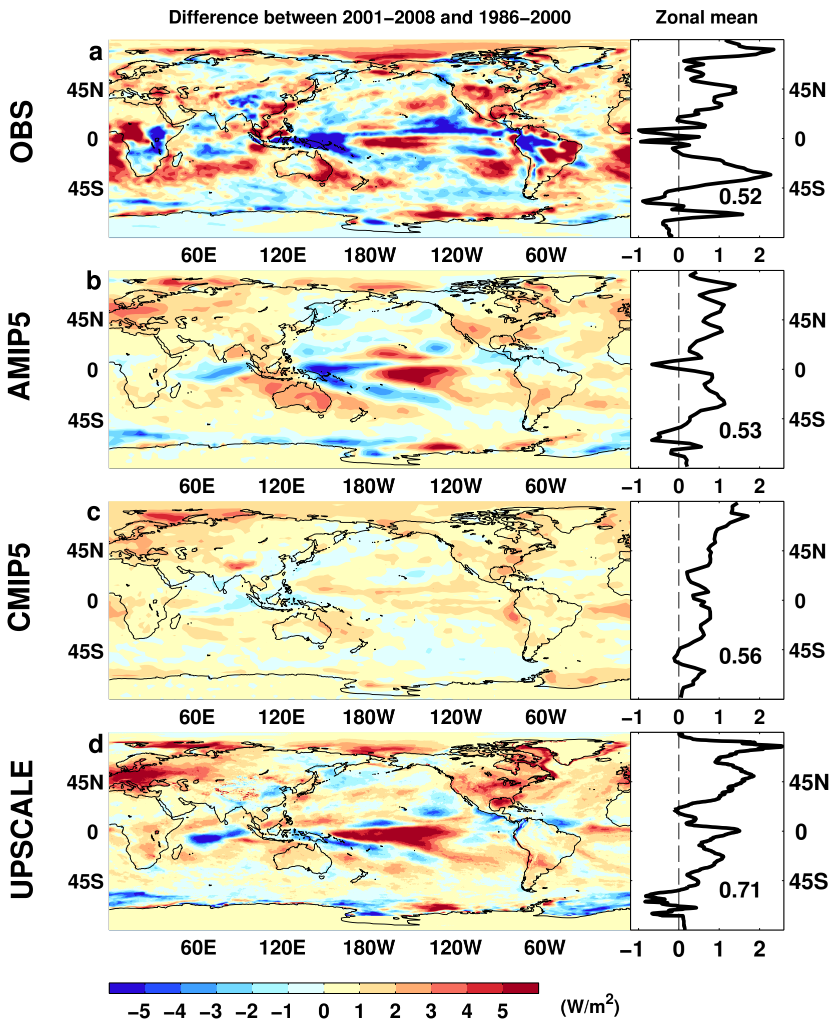

Figure S 6. Change in absorbed solar radiation (Wm^{-2}) 2001–2008 minus 1986–2000 for (a) OBS, (b) AMIP5, (c) CMIP5 and (d) UPSCALE. Global mean values are displayed in zonal mean plots.

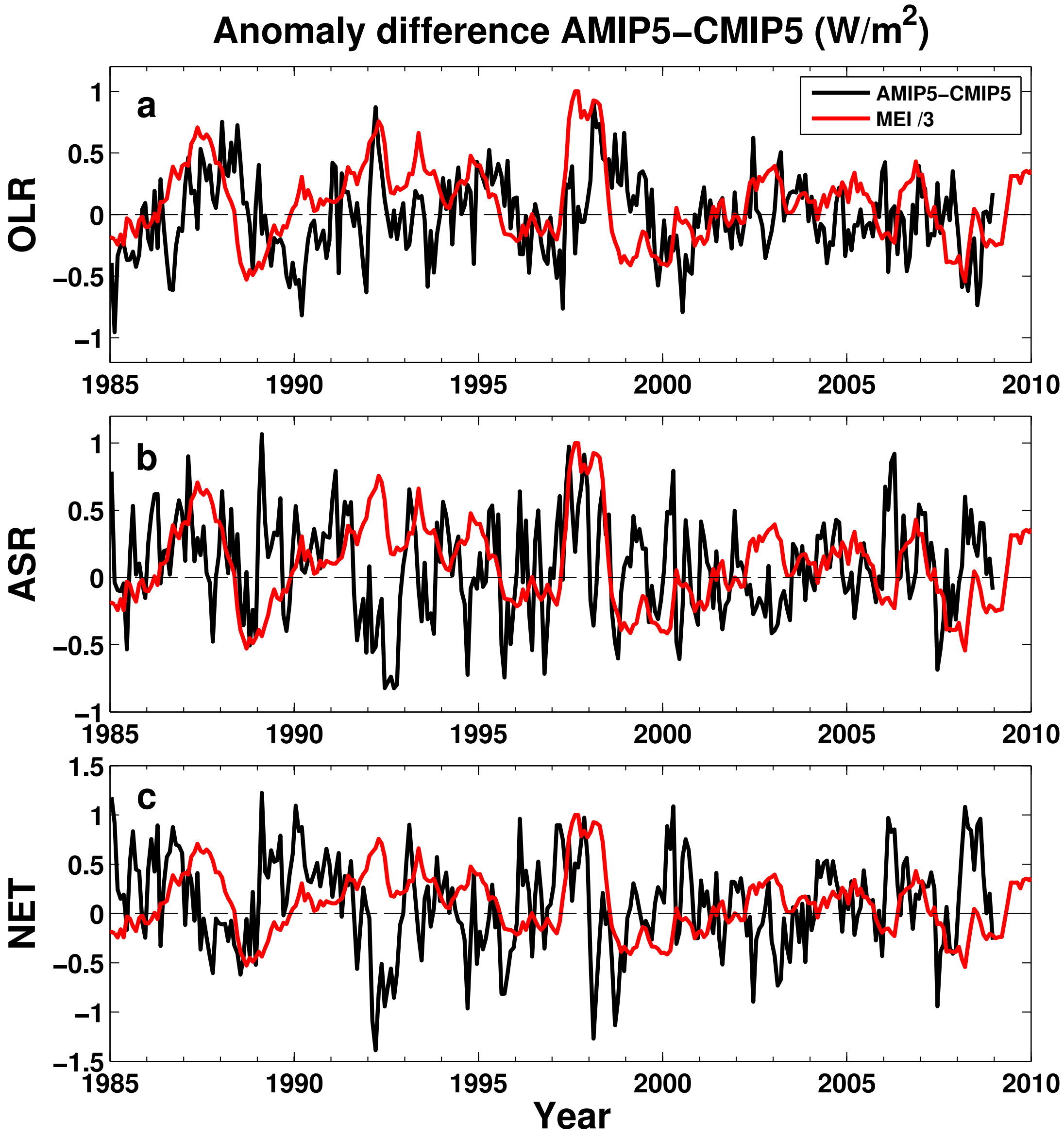

Figure S 7. AMIP5 minus CMIP5 ensemble mean top of atmosphere (a) outgoing longwave radiation, (b) absorbed solar radiation and (c) net downward radiation and a Multivariate ENSO index (values divided by 3).

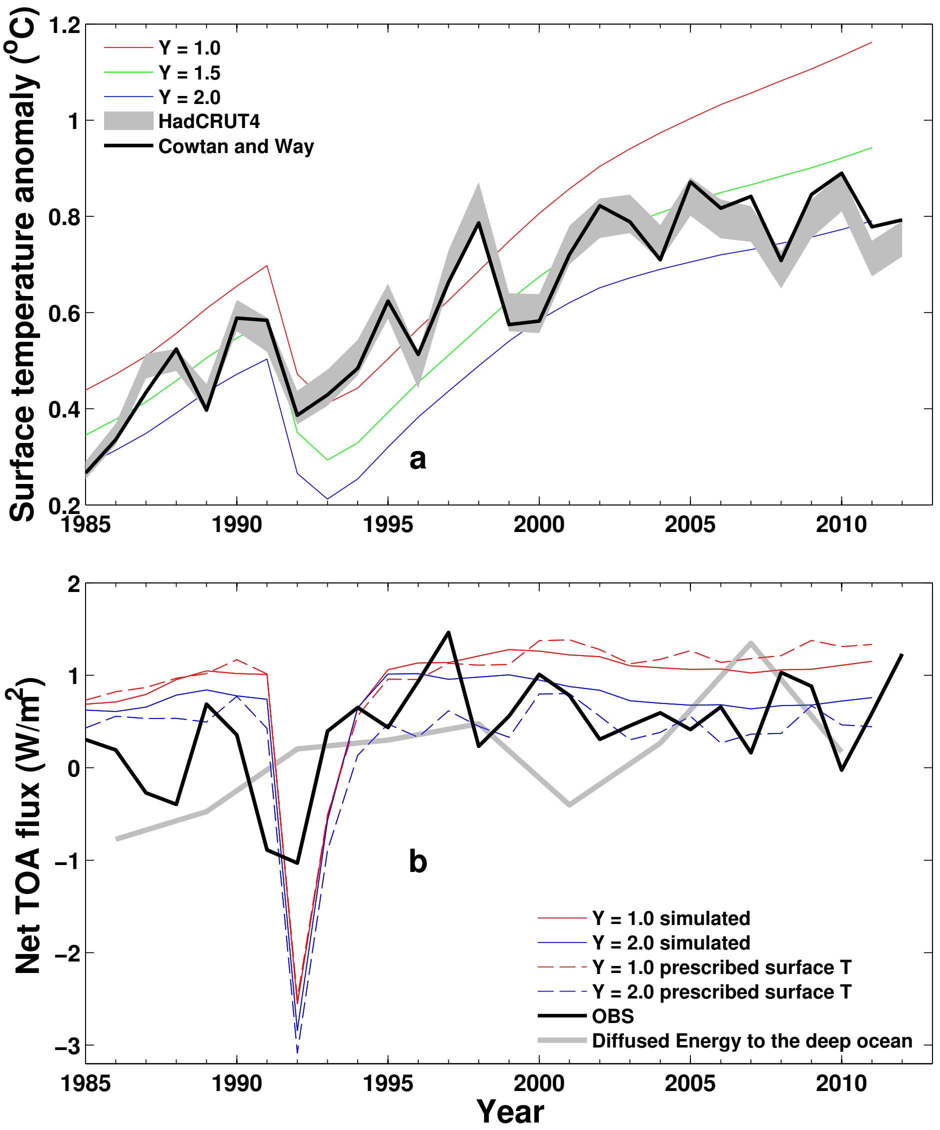

Figure S 8. (a) Global annual average surface temperature change relative to 1850-1870 from HadCRUT4 observations including an adjustment by *Cowtan and Way* [2013] and simulated by the simple model with prescribed radiative forcing and varying climate feedback parameter, Y ($Wm^{-2}K^{-1}$). (b) Net top of atmosphere imbalance from OBS and simulated by the simple model (using computed and prescribed T_s) and inferred changes in heat flux below the ocean mixed layer (3-year averages)

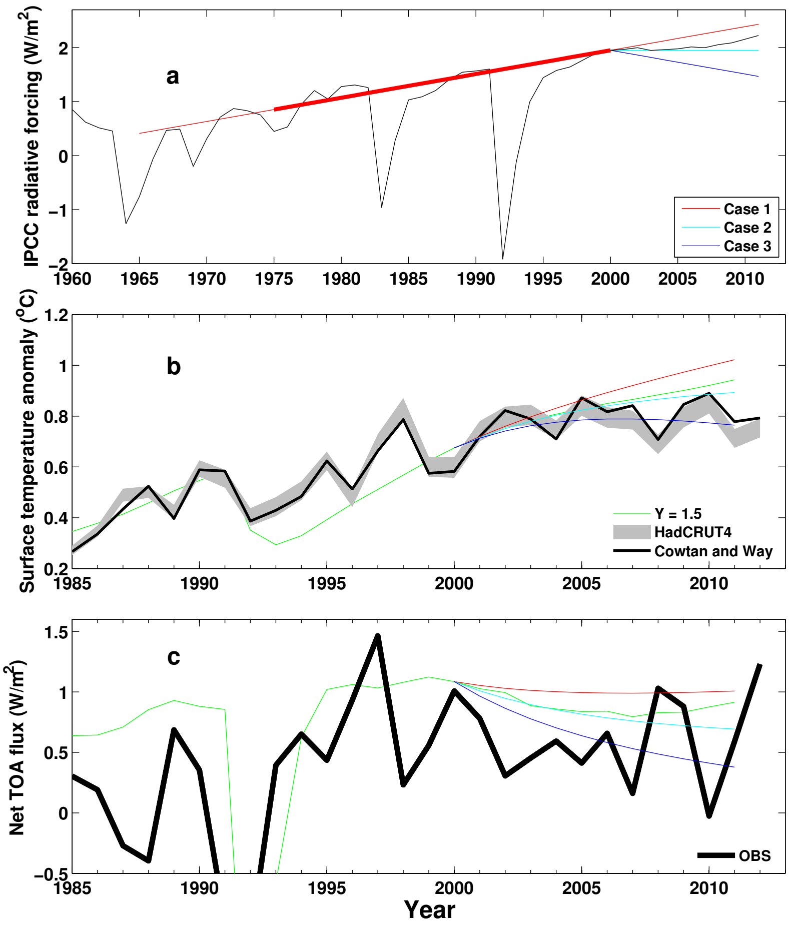

Figure S 9. Influence of trends in radiative forcing on surface temperature (T_s) and top of atmosphere net radiative imbalance (N) during the 2000s: (a) changes in radiative forcing (ΔF) relative to 1860 (black), the trend in ΔF 1975-2000 (red line) without including years influenced by volcanic forcing (1982-1984 and 1991-1993) and idealized changes in ΔF from 2000; a continuation of the 1975-2000 trend (case 1: thin red), constant ΔF (case 2: cyan) and a negative trend of the same magnitude as case 1 (case 3: blue); (b) changes in T_s from HadCRUT4 (gray shading), HadCRUT4 with *Cowtan and Way* [2013] adjustment (black) and simulated by the simple model (green) and for each case in (a) with corresponding colors; (c) changes in N from OBS (black) and simulated by the simple model (green) and for each case in (a) with corresponding colors.
